# Exploring the Activity Profile of TbrPDEB1 and hPDE4 Inhibitors Using Free Energy Perturbation

**DOI:** 10.1021/acsmedchemlett.1c00690

**Published:** 2022-05-23

**Authors:** Lorena Zara, Francesca Moraca, Jacqueline E. Van Muijlwijk-Koezen, Barbara Zarzycka, Robert Abel, Iwan J. P. de Esch

**Affiliations:** † Amsterdam Institute of Molecular and Life Sciences (AIMMS), Division of Medicinal Chemistry, Faculty of Science, Vrije Universiteit Amsterdam, De Boelelaan 1108, 1081 HZ Amsterdam, The Netherlands; ‡ Schrodinger, Inc., 1540 Broadway, New York, New York 10036, United States

**Keywords:** Free energy perturbation, Binding free energy, Phosphodiesterases

## Abstract

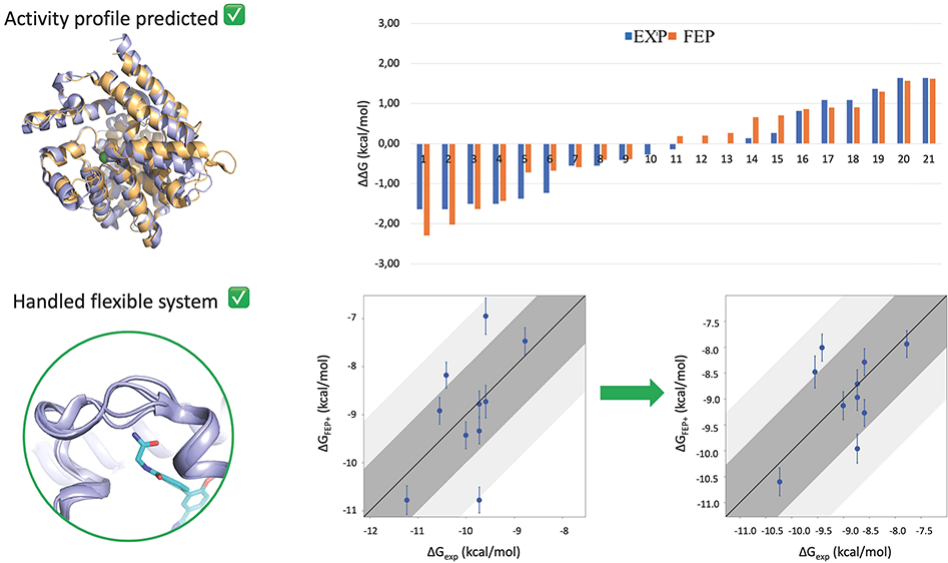

Human African trypanosomiasis (HAT) is a neglected tropical disease caused by the parasite *Trypanosoma brucei* (*T.b*.). A validated target for the treatment of HAT is the parasitic *T.b.* cyclic nucleotide phosphodiesterase B1 (TbrPDEB1). Although nanomolar TbrPDEB1 inhibitors have been obtained, their activity against the off-target human PDE4 (hPDE4) is likely to lead to undesirable clinical side effects, such as nausea, emesis, and immune suppression. Thus, new and more selective TbrPDEB1 inhibitors are still needed. This retrospective study evaluated the free energy perturbation (FEP+) method to predict the affinity profiles of TbrPDEB1 inhibitors against hPDE4. We demonstrate that FEP+ can be used to accurately predict the activity profiles of these homologous proteins. Moreover, we show how FEP+ can overcome challenges like protein flexibility and high sequence conservation. This also implies that the method can be applied prospectively for the lead optimization campaigns to design new and more selective TbrPDEB1 inhibitors.

Human African trypanosomiasis (HAT), commonly known as African sleeping sickness, is a neglected tropical disease caused by the parasite *Trypanosoma brucei* (*T.b*.), a protozoan transmitted through the bite of a tsetse fly to humans. The disease is fatal when left untreated.^1^ The range of drugs used against it is limited, and the current treatments often show resistance toward the parasite and cause severe toxicity to humans.^2^ Therefore, the exploration of new safe drugs for HAT remains a critical medical need.

There are four parasite PDEs (TbrPDE A-D) that play a vital role in the life cycle of the parasite as they catalyze the hydrolysis of cyclic adenosine monophosphate (cAMP) and (to a lesser extend) cyclic guanosine monophosphate (cGMP) to AMP and GMP, respectively.^3^ Genetic knockout studies using RNAi have shown that the cyclic nucleotide phosphodiesterase B1 (TbrPDEB1) is a promising therapeutic target for HAT.^4,5^ The early TbrPDEB1 inhibitors most often have even higher activity for hPDE4, which in clinical applications might lead to undesirable side effects, such as nausea, emesis, and immune suppression.^6−10^ Considering function and sequence identity, the TbrPDEB1 binding site is the most similar to the human PDE4 (hPDE4) and has 27% and 35% sequence identity^11^ with the hPDE4B and hPDE4D isoforms (Figure S1).

The known TbrPDEB1 inhibitors interact with the substrate (cAMP) binding site, in which the aromatic rings of the substrate and the inhibitors are positioned in a hydrophobic clamp (HC) formed by Phe887^HC.52^ and Val840^HC.32^. Hydrogen bonding with the conserved glutamine Gln874^Q.50^ places the substrate in such a way that the two metal ions can catalyze substrate conversion. The conserved glutamine and the HC form the so-called Q pocket (Figure 1 and Figure S2). While all PDE enzymes contain these structural elements, the TbrPDEB1 structure has a unique pocket, absent in the 11 human PDEs. The parasite PDEs contain a highly flexible pocket, as demonstrated by the high B-factor, formed by the M-loop, helix 14 (H14), and helix 15 (H15) (Figure 1b). This pocket is absent in the 11 human PDEs and therefore is considered as an opportunity to develop selective TbrPDEB1 inhibitors^12^ (Figure 1a and 1b). Other parasite PDEs, e.g., *Leishmania major* PDEB1 (LmjPDEB1), also have such a pocket which is therefore called the parasite-specific pocket or P-pocket.^13^

**Figure 1:**
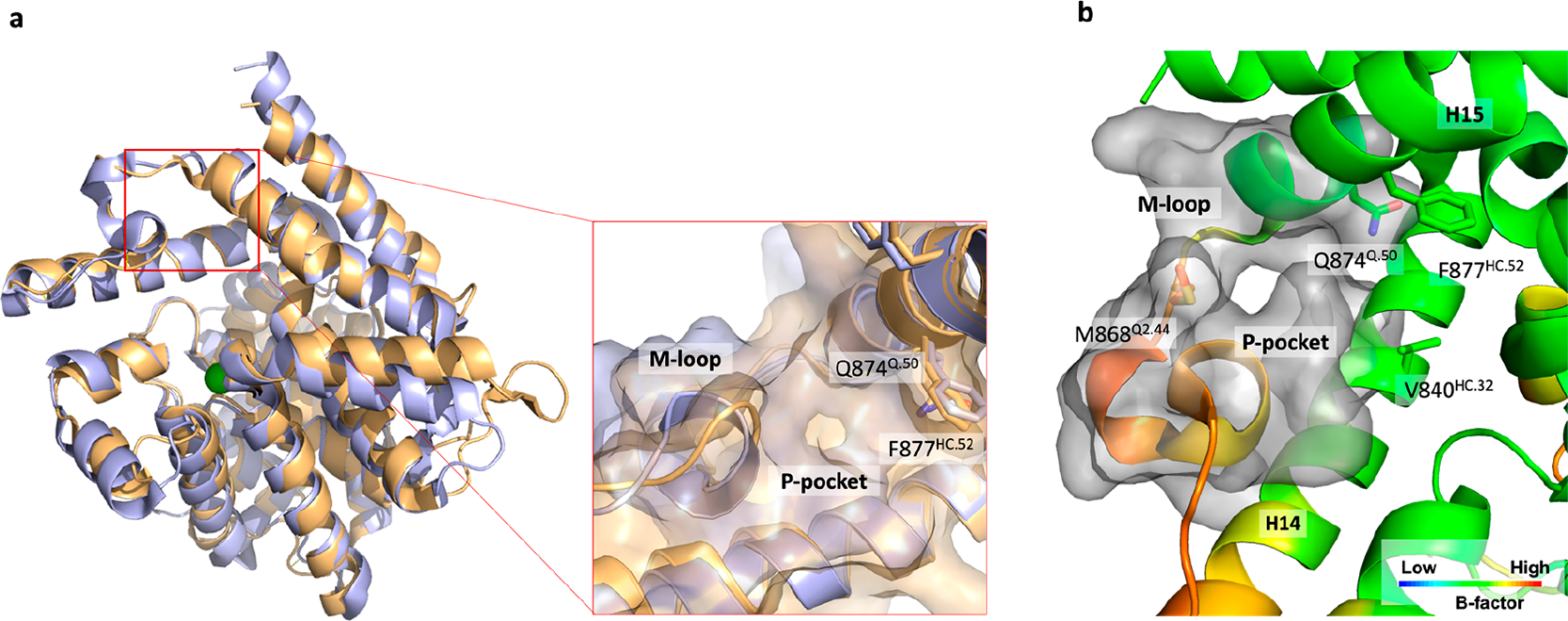
(a) Structure comparison of hPDE4D (PDB ID: 6HWO) and TbrPDEB1 (PDB ID: 6GXQ). The specific P-pocket in TbrPDEB1 is shown as a blue surface, while the same pocket is not present in the hPDE4D protein structure (orange surface). (b) Zoom of the TbrPDEB1 binding site with the carbon atoms colored according to the B-factor (PDB ID: 4I15), and the P-pocket is shown as the gray surface. All binding site residues have been named according to the PDEStrIAn nomenclature convention.^14^

The TbrPDEB1 P-pocket is successfully probed by a series of compounds that contain a tetrahydrophthalazinone scaffold, leading to inhibitors with higher activity for TbrPDEB1 than for hPDE4 enzymes.

Crystal structures confirm these inhibitors interact with the P-pocket with various polar groups (Figure 2a).^12^ These structures also confirm the flexibility of the P-pocket, especially by the movement of the M-loop. While the P-pocket gives obvious opportunities to obtain TbrPDEB1 selectivity, a few specific compound series that merely interact with the substrate-binding site that is conserved in all PDE enzymes also offer improved activity profiles, e.g., the alkynamide-phthalazinone series^15^ inhibits TbrPDEB1 slightly better than hPDE4B1.

**Figure 2:**
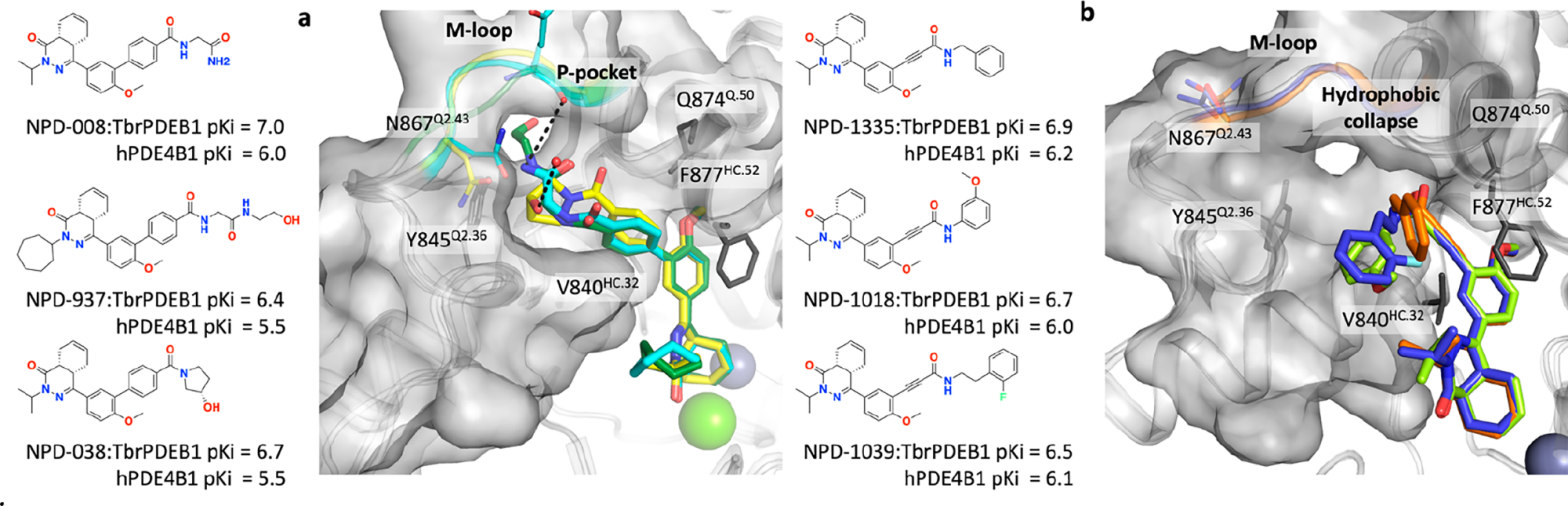
(a) Superimposition of tetrahydrophthalazinone TbrPDEB1 inhibitors binding into the P-pocket: NPD-008 (cyan carbon atoms), NPD-937 (green carbon atoms), NPD-038 (yellow carbon atoms) (PDB IDs: 5G2B, 5L8Y, 5G5V, respectively). (b) Superimposition of alkynamide phthalazinone TbrPDEB1 inhibitors: NPD-1335 (orange carbon atoms), NPD-1018 (green carbon atoms), NPD-1039 (violet carbon atoms) (PDB IDs: 6GXQ, 6RFN, 6RFW, respectively). Key binding site residues are shown as sticks; the water molecule interacting with NPD-038 is shown as a sphere, and the rest of the water molecules are omitted for clarity. Zinc and magnesium cations are displayed respectively as metallic blue and green spheres. P-pocket residues Ala837^Q1.30^, Thr841^Q2.33^, Tyr845^Q2.36^, Asn867^Q2.43^, Met868^Q2.44^, Glu869^Q2.45^, and Leu870^Q2.46,^ are shown as molecular surfaces. All binding site residues have been named according to the PDEStrIAn nomenclature convention.^14^

TbrPDEB1 ligands from this class adopt a conformation that has been described as a hydrophobic collapse, and the ligands do not interact with the P-pocket in TbrPDEB1.^16^ The lack of interaction with this flexible region avoids ligand-induced conformational changes of the P-pocket, as the crystal structures, shown in Figure 2b, demonstrate. Intriguingly, the binding mode of the alkynamide-phthalazinone does not provide a clear explanation for the improved activity profile.^16^ In fact, the ligand adopts an identical binding mode characterized by a bidentate interaction with residue Gln874^Q.50^ in both TbrPDEB1 and hPDE4D (PDB IDs: 6GXQ and 6HWO, respectively) (Figure S2).

This study evaluates whether free energy perturbation (FEP+) can retrospectively predict the TbrPDEB1-hPDE4 activity profile for the two series (tetrahydrophthalazinones and alkynamide-phthalazinone). It was previously shown that the FEP+ approach could accurately predict the selectivity profiles of inhibitors for pairs of human PDEs (i.e., hPDE9A-PDE1C and hPDE5A-6C).^17^ While the different classes of TbrPDEB1-hPDE4 ligands have similar activity profiles, the structural data is expected to represent different challenges for the implementation of accurate FEP+ calculations (i.e., the flexibility of the P-pocket and the similarity of the HC region). Dynamic binding sites have, in fact, shown to be challenging for free energy methods.^18,19^ We investigated the stability and flexibility of different TbrPDEB1 complexes by running 100 ns molecular dynamics (MD) simulations with the Desmond simulation package. The obtained MD trajectories were used to calculate the root-mean-square deviation (RMSD) of the ligands during the simulations, and considerable movements of the inhibitors were found (up to 3.5 Å). The trajectory analysis showed how the ligand core maintains the key interactions with the protein throughout the simulation, while the R-group pointing to the flexible P-pocket is the portion of the ligand causing the high RMSD. Only the structure of TbrPDEB1 in the complex with NPD-008 (PDB ID: 5G2B) showed good ligand stability during the MD trajectory, with an RMSD of 1.8 Å. This is probably due to the key hydrogen bond (HB) the ligand is forming with the residues in the P-pocket. This structure was therefore selected as the input structure for calculating the activity profile of tetrahydrophthalazinone compounds in TbrPDEB1.

The tetrahydrophthalazinone compounds were split into two validation sets according to the similarity of the ligand R-group pointing to the P-pocket; this enables us to identify potential differences in accuracy for predictions in this region. The first set (set 1) comprises compounds characterized by an alkyl chain as the R1-group pointing in the direction of the P-pocket (Table S1). In contrast, set 2 contains compounds characterized by an aliphatic 5-membered ring in the R1-group (Table S2). Glide was used to dock the compounds into the TbrPDEB1 binding site (PDB ID: 5G2B), and then full-cycle FEP+ calculations were performed with default settings.

The accuracy of the free energy calculations for set 1 and set 2 was assessed by calculating the mean unsigned error (MUE) and the root-mean-square error (RMSE) resulting respectively in 1.10 kcal/mol ± 0.2 and 1.45 kcal/mol ± 0.3 for set 1 and 1.70 kcal/mol ± 0.2 and 1.88 kcal/mol ± 0.3 for set 2. Further information on the data sets can be found in Table S4. Compared to accuracies reported in an earlier benchmark set^20^ and drug discovery projects at Schrödinger,^21^ these results, especially for set 2, show a low accuracy of our initial calculations. In fact, free energy estimation is appealing for drug optimization when RMSE is smaller than 1.3 kcal/mol.^22,23^

We hypothesized that the low accuracy of the initial calculations might be caused by the insufficient sampling of the flexible M-loop, part of the P-pocket region. Previous studies^24^ have shown how the inclusion of portions of both the ligand and protein in the replica exchange with solute tempering (REST) region^25^ can be used to address the sampling issue, leading to significant improvements in the FEP+ prediction.^26^

Therefore, we repeated the FEP+ calculation by extending the part of the ligand included in the REST region by using the custom core constraints to increase ligand sampling. Since the results did not improve, we also included the Thr841^Q2.33^ side chain in the REST region to check if the prediction inaccuracy would come from the overestimation of the HB between Thr841^Q2.33^ and the NH of the phenyl-amide moiety shared among all the ligands. This led to substantial improvements in set 1, with a decrease in the number of outliers (no edges were predicted with a ΔΔ*G* error > 3 kcal/mol) (Figure 3c). Furthermore, the accuracy of calculations over set 1 drastically improved, resulting in the RMSE vaue of 1.03 kcal/mol ± 0.1 and the MUE value of 0.86 kcal/mol ± 0.1.

**Figure 3:**
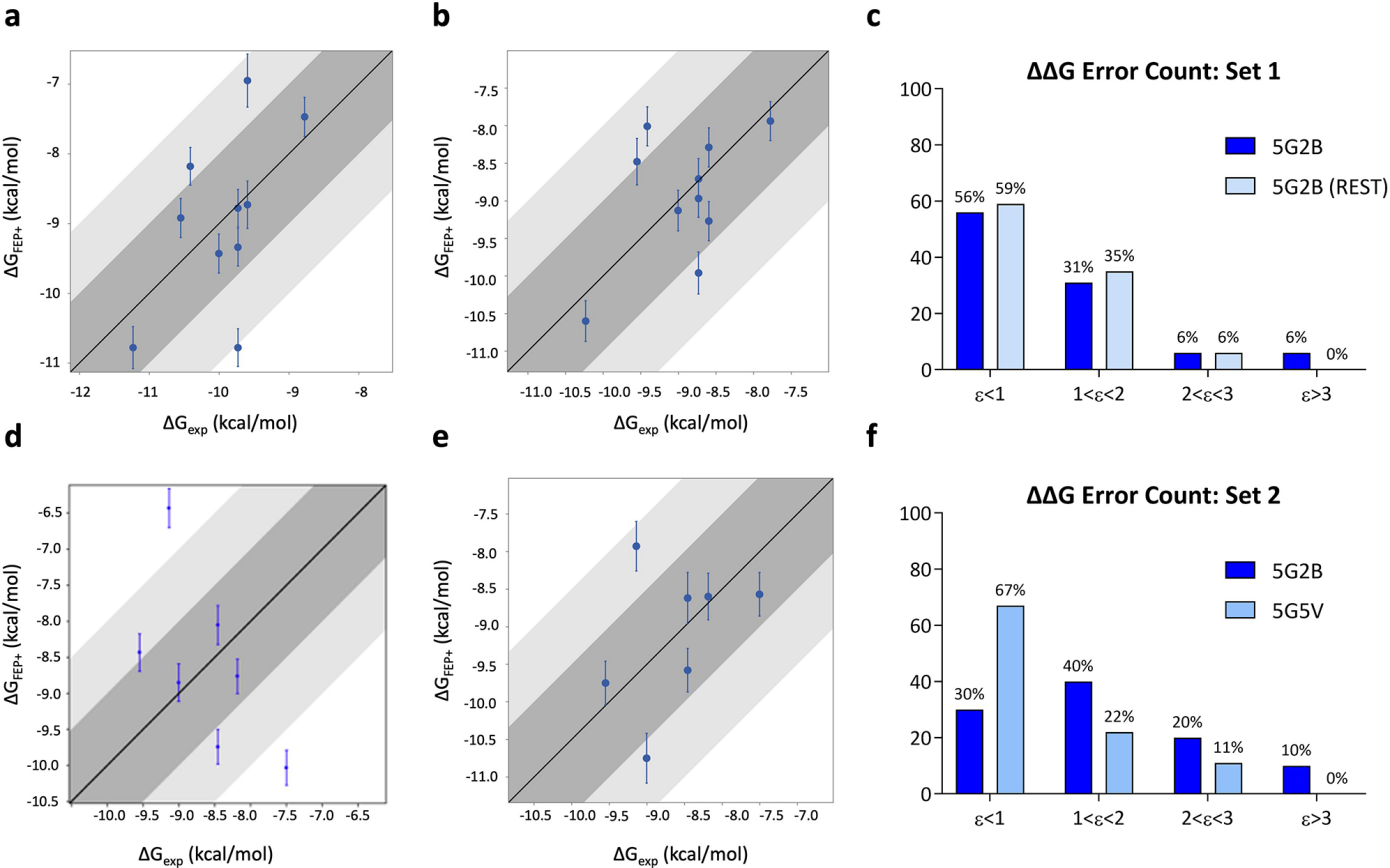
Correlation plots between experimental (*X*-axis) and predicted (*Y*-axis) binding affinities for tetrahydrophthalazinone ligands calculated for TbrPDEB1 of set 1 (a) (PDB ID: 5G2B) with default parameters and (b) adding to the REST region Thr841^Q2.33^ and of set 2 (d) (PDB ID: 5G2B) and (e) (PDB ID: 5G5V). ΔΔ*G* error count for (c) set 1 and (f) set 2 against TbrPDEB1. Bin 1 corresponds to a ΔΔ*G* error ≤ 1 kcal/mol; bin 2 corresponds to 1 < ΔΔ*G* error ≤ 2 kcal/mol; bin 3 corresponds to 2 < ΔΔ*G* error ≤ 3 kcal/mol; bin 4 corresponds to a ΔΔ*G* error > 3 kcal/mol.

For set 2, the results did not improve using the custom core constraint nor the protein extended REST region. For this reason, we decided to repeat the FEP+ calculation for set 2 using a different reference crystal structure. The newly selected structure (PDB ID: 5G5V) is crystallized in complex with NPD-038. This ligand, included in set 2, shares the same core as NPD-008 but is characterized by the rigidified aliphatic 5-membered ring in the R1-group (as the other ligands in set 2).

The better performance indicates the dependency of the method on the crystal structure used as input for calculation.

The change of reference structure led to an improvement in the accuracy, with respective MUE and RMSE values of 0.94 kcal/mol ± 0.2 and 1.13 kcal/mol ± 0.3. Significantly, all edges were predicted with less than 3 kcal/mol ΔΔ*G* error (i.e., no outliers), while improving the number of edges predicted with less than 1 kcal/mol ΔΔ*G* error to 67% (Figure 3f).

When the ligand is in contact with a flexible region, the initial conformation of the protein structure used affects the accuracy of the calculations.^22,27,28^ In these cases, the alignment can differ substantially also for chemically similar compounds from the same congeneric series.

The FEP+ calculations performed using sets 1 and 2 on the human target were more straightforward. Notably, the choice of a common input structure (PDB ID: 5LAQ) for both sets of compounds did not affect the accuracy of the results, as opposed to the TbrPDEB1 calculations. In fact, for hPDE4, the results were satisfying for both sets of ligands, with MUE and RMSE values respectively of 0.69 kcal/mol ± 0.1 and 0.90 kcal/mol ± 0.2 for set 1 and of 0.64 kcal/mol ± 0.1 and 0.79 kcal/mol ± 0.1 for set 2. The number of edges with a ΔΔ*G* error > 3 kcal/mol was 0, for both sets 1 and 2 (Figure 4). This might be explained by the fact that hPDE4 does not have the flexible P-pocket, and ligands bind to a more rigid region of the binding site; therefore, there are no sampling issues.

**Figure 4:**
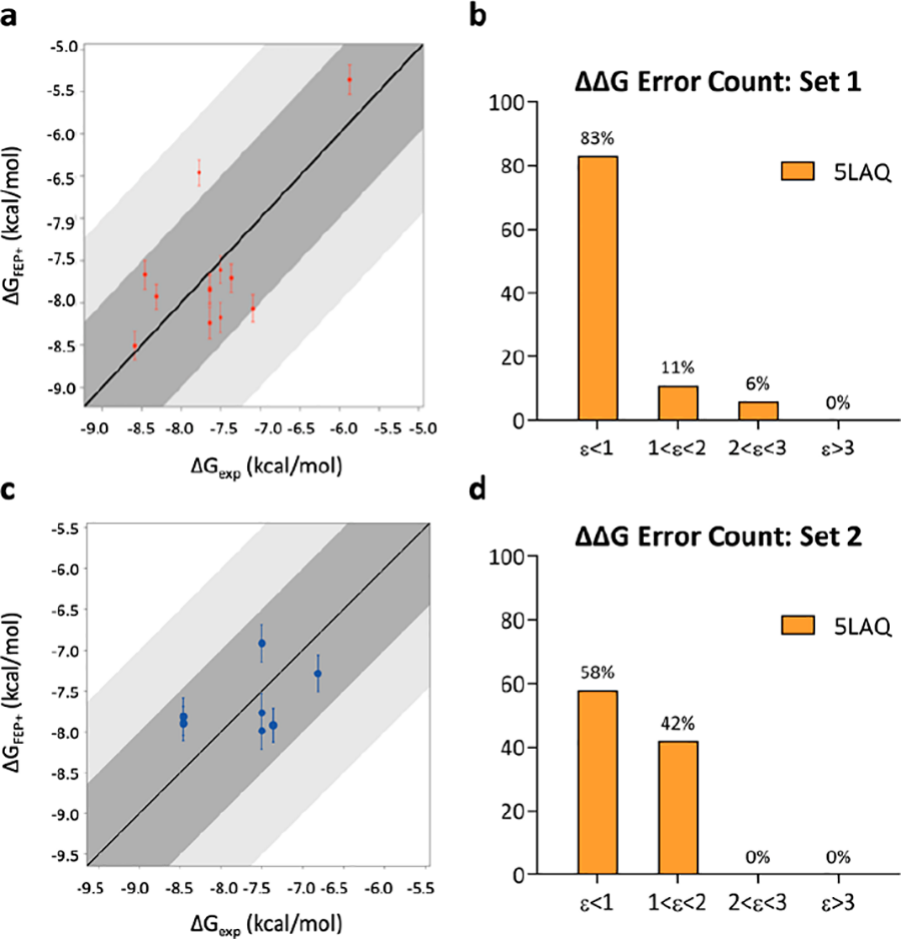
Correlation plots between experimental (*X*-axis) and predicted (*Y*-axis) binding affinities for tetrahydrophthalazinone ligands of (a) set 1 and (c) set 2 were calculated for hPDE4B (PDB ID: 5LAQ). (b) ΔΔ*G* error count for set 1 and (d) for set 2. Bin 1 corresponds to a ΔΔ*G* error ≤ 1 kcal/mol; bin 2 corresponds to 1 < ΔΔ*G* error ≤ 2 kcal/mol; bin 3 corresponds to 2 < ΔΔ*G* error ≤ 3 kcal/mol; bin 4 corresponds to a ΔΔ*G* error > 3 kcal/mol.

The second class of ligands investigated by FEP+ is the alkynamide phthalazinones, a potent class of TbrPDEB1 inhibitors. These compounds, different from the tetrahydrophthalazinones, do not target the P-pocket.^15,16^ Instead, their R-groups fold back toward the Phe877^HC.52^ of the conserved HC in the so-called hydrophobic collapse (Figure 2b).

For the study of this series (set 3) (Table S3), we selected as the input structure PDB ID: 6GXQ, i.e., TbrPDEB1 in complex with NPD-1335. This compound is the most potent (p*K*_i_ = 6.8) from this series and is cocrystallized in both parasite and human PDEs.^16^

The structural diversity of the ligands in the congeneric series is represented by R1 in Table S3; those were docked with Glide and then used to run FEP+ prediction at default settings. Calculations resulted in high prediction accuracy with MUE and RMSE values of 0.89 kcal/mol ± 0.1 and 1.08 kcal/mol ± 0.1, respectively. Notably, all the edges were predicted with a ΔΔ*G* error < 3 kcal/mol (Figure 5b).

**Figure 5:**
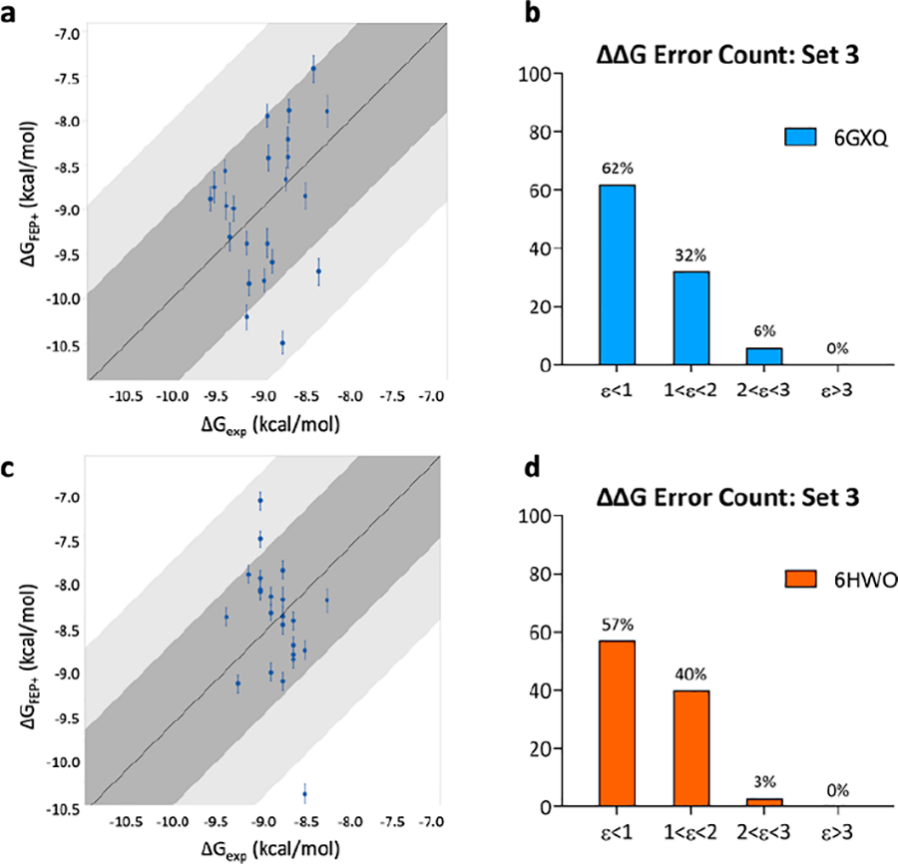
Correlation plots between experimental (*X*-axis) and predicted (*Y*-axis) binding affinities for alkynamide phthalazinone ligands of set 3 calculated for (a) TbrPDEB1 (PDB ID: 6GXQ) and (c) hPDE4D (PDB ID: 6HWO). (b) ΔΔ*G* error count for set 3 against TbrPDEB1 and (d) hPDE4D. Bin 1 corresponds to a ΔΔ*G* error ≤ 1 kcal/mol; bin 2 corresponds to 1 < ΔΔ*G* error ≤ 2 kcal/mol; bin 3 corresponds to 2 < ΔΔ*G* error ≤ 3 kcal/mol; bin 4 corresponds to a ΔΔ*G* error > 3 kcal/mol.

The FEP+ predictions on the alkynamide phthalazinones resulted in higher accuracy than the tetrahydrophthalazinone class.

We hypothesized that the lower ligand-induced fit observed with this class of compounds could be the reason for the better performance of the FEP+ method. Since they do not interact with the P-pocket residues, their binding does not affect the conformation of the flexible M-loop. This can be observed by the superimposition of different TbrPDEB1 crystal structures binding with alkynamide phthalazinones, where the variability on the M-loop conformation is very low (Figure 2b). This was confirmed by measuring the RMSD values of the P-pocket residues with respect to the apo-structure (PDB ID: 4I15). The analysis showed a low degree of induced fit of the P-pocket residues (RMSD < 1 Å). In contrast, the same analysis performed using the TbrPDEB1 structures cocrystallized with different tetrahydrophthalazinones showed a much higher variability on this region (RMSD > 3 Å), proving the induced fit effect upon binding of the P-pocket binders (Table S5).

As expected, the HC residues that are part of helix H14 present lower flexibility than the M-loop residues. The binding of alkynamide phthalazinones does not lead to an induced fit of the TbrPDEB1 binding site. The different accuracies obtained across the two classes of compounds enforce the idea that interactions with the flexible part of the system, i.e., the P-pocket, pose a sampling challenge for FEP+.

For the retrospective application of FEP+ to set 3 against hPDE4, we selected PDB ID: 6HWO as the input structure of the hPDE4D enzyme in complex with NPD-1335. Interestingly, this inhibitor binds in an almost identical manner to both catalytic sites of hPDE4D and TbrPDEB1, maintaining the key hydrophobic interactions in the HC region and a bidentate HB with the conserved Gln874^Q.50^ (Figure S2).

The calculations resulted in high accuracy between computed and experimental Δ*G*, with MUE and RMSE values of 0.82 kcal/mol ± 0.1 and 1.00 kcal/mol ± 0.1, respectively. Furthermore, the edges predicted with a ΔΔ*G* error > 3 kcal/mol were 0 (Figure 5d). The results obtained in the two species are comparable, with high accuracy between experimental and predicted Δ*G*.

Finally, we built the activity correlation plots (Figure 6) to evaluate if FEP+ could correctly predict the activity profiles in the PDEs pair. The selectivity is shown by the deviation of the bars from zero. Interestingly, for set 1, the FEP+ calculations seem to overemphasize the selectivity on either side (Figure 6a), while for set 2, the profiles that are calculated with FEP+ are more in line with the experimental data, as can be noticed in Figure 6b where the blue and orange bars show the same trend indicating an agreement between experimental and computed affinities in both targets.

**Figure 6:**
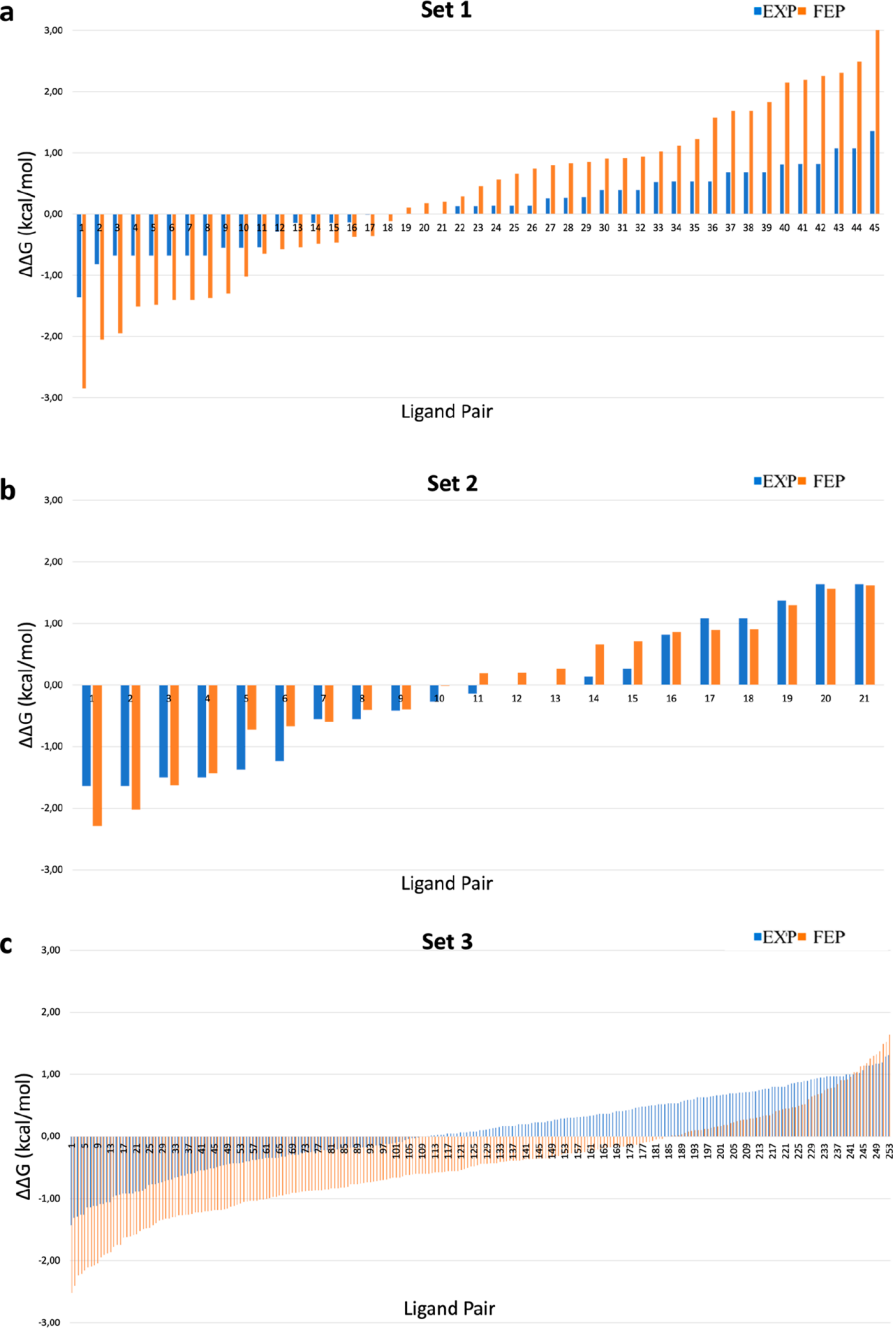
TbrPDEB1/hPDE4D activity correlation plots for sets 1, 2, and 3, with the ligand pairs presented on the *X*-axis. The *Y*-axis represents the selectivity, expressed by the differences of their Δ*G* in TbrPDEB1-hPDE4D (ΔΔ*G*_s_ = Δ*G*_TbrPDEB1_ – Δ*G*_hPDE4D_). The blue bars represent the experimental values, and the orange bars represent the predicted values by FEP+: (a) set 1, (b) set 2, and (c) set 3.

For set 3, the activity correlation plot (Figure 6c) shows great agreement with the experimental values, with the experimental activity range between −1.4 and +1.4 (blue line) and the predicted activity range between −2.6 and +1.7 (orange lines).

In conclusion, our study shows the successful application of free energy perturbation for the retrospective prediction of the activity profiles for the target enzyme TbrPDEB1 and the homologous off-target hPDE4. Notably, we show that FEP+ calculations can retrospectively predict the relative binding energies with a high level of accuracy for both flexible and conserved parts of the binding sites.

Moreover, our study, in accordance with previously reported cases,^29,30^ demonstrates the significance of the thorough selection of the input structure used for the FEP+ calculation as well as the set of ligands, especially for flexible systems where ligands determine an induced fit on the binding site.

This retrospective validation study suggests that tetrahydrophthalazinone and alkynamide phthalazinone classes, targeting distinct parts of the binding pocket (the P-pocket and the HC), constitute an amenable system for the FEP+ calculation to follow up in the prospective lead optimization campaign to design new optimized and more selective TbrPDEB1 inhibitors.

## Supporting Information Available

Materials and methods; sequence alignment of hPDE4D, hPDE4B, and TbrPDEB1; interaction fingerprints and 2D ligand graphs; Glide SP score, Δ*G* values experimentally determined and respective Δ*G* computed by FEP+ for sets 1, 2, and 3; statistics on data sets used for FEP+ calculations; flexibility and stability analysis; and convergence data from FEP+ calculations (PDF)

## Abbreviations

PDE: phosphodiesterases
TbrPDEB1: *Trypanosoma brucei* PDE B1
hPDE4: human PDE 4
cAMP: cyclic adenosine monophosphate
cGMP: cyclic guanosine monophosphate
FEP: free energy perturbation
REST: replica exchange with solute tempering
RMSE: root-mean-square error
MUE: mean unsigned error
MD: molecular dynamics
P-pocket: parasite-specific pocket
HC: hydrophobic clamp.
