# Supplementary material for: Exploring the Activity Profile of TbrPDEB1 and hPDE4 Inhibitors Using Free Energy Perturbation
Source: ACS Med Chem Lett. 2022 May 23;13(6):904–10. doi: 10.1021/acsmedchemlett.1c00690 (PMC9190044; doi:10.1021/acsmedchemlett.1c00690)
Supplement: Supplementary file 1 — ml1c00690_si_001.pdf [file ml1c00690_si_001.pdf]

SUPPORTING INFORMATION

Exploring the activity profile of TbrPDEB1 and hPDE4 inhibitors using Free Energy Perturbation (FEP+)

Lorena Zara[1], Francesca Moraca[2], Jacqueline E. Van Muijlwijk-Koezen[1], Barbara Zarzycka[1], Robert Abel[2], Iwan J.P. de Esch[1*]

[1]Amsterdam Institute of Molecular and Life Sciences (AIMMS), Division of Medicinal Chemistry, Faculty of Science, Vrije Universiteit Amsterdam, De Boelelaan 1108, 1081 HZ Amsterdam, The Netherlands

[2]Schrodinger, Inc. 1540 Broadway, NY 10036, USA

Table of content

Table of content 2

Materials and methods 3

Ligand Preparation........ 3

Protein Structure Preparation........ 3

Ligand docking 4

FEP+ calculation........ 4

Figure S1: Sequence alignment of human PDE4D, PDE4B, and parasite TbrPDEB1, colored by identity........ 5

Figure S2. Interaction fingerprints and 2D ligand graphs........ 6

Table S1. Chemical structures of tetrahydrophthalazinones ligands of validation set 1, Glide SP score, ΔG values experimentally determined and respective ΔG computed by FEP+....... 10

Table S2. Chemical structures of tetrahydrophthalazinones ligands of validation set 2, Glide SP score, ΔG values experimentally determined and respective ΔG computed by FEP+....... 11

Table S3. Chemical structures of alkynamide phthalazinones ligands of validation set 3, Glide SP score, ΔG values experimentally determined and respective ΔG computed by FEP+....... 12

Table S4. Statistics on Data Sets used for FEP+ calculations........ 14

[a]Thr841 included in the REST region 14

Table S5. Flexibility and stability analysis of the X-ray structures in complex with PDEs inhibitors........ 14

Table S6. Convergence data from FEP+ calculation........ 15

Figure S3: Examples of Good, Fair, and Bad convergence plots and the associated maps.... 16

References........ 18

Materials and methods

This work aims to validate the use of FEP+ to assess the activity profiles of multiple series of TbrPDEB1 inhibitors against the off-target hPDE4. The flexibility of the specific P-pocket in TbrPDEB1 constituted the challenge for FEP+ calculations. We considered it essential to analyze the degree of induced fit of the different ligand classes upon binding to address this issue.

Ligand Preparation

Data sets were collected from an in-house database (SI Table 1, 2, and 3). All PDE inhibitors considered in this study were built using the Maestro 19 interface[1]; tautomer enumeration and protonation state assignment at experimental pH of 7.0 +/- 2 was performed using LigPrep[2].

Protein Structure Preparation

All protein structures were obtained from the Protein Data Bank (PDB) (www.rcsb.org) and imported into Maestro. The tetrahydrophtalazinone series against TbrPDEB1 was investigated using the X-rays (PDB IDs: 5G57, 1.73 Å resolution, 5G2B, 1.83 Å resolution)[3] in complex with NPD-038 and NPD-008, respectively. No ligands belonging to the tetrahydrophtalazinone series have nowadays been co-crystallized with any hPDE4. Therefore hPDE4B (PDB ID: 5LAQ, 2.40 Å resolution) and hPDE4D (PDB ID: 5LBO, 2.25 Å resolution) [3] in complex with NPD-001 were investigated.

For the analysis of the alkynamide phtalazinone series TbrPDEB1 (PDB ID: 6GXQ, 1.96 Å resolution) and hPDE4D (PDB ID: 6HWO, 1.99 Å resolution), [4] in complex with NPD-1335, were selected.

The structures were prepared using the Protein Preparation Wizard[5,6] tool in Maestro. Hydrogen atoms were added, missing side chains were filled, all crystal waters were retained.

The proper ionization state was assigned for both the amino acids and co-crystallized ligand at physiological pH.

The stability of the co-crystallized ligands in their corresponding binding site was assessed by running 100 ns MD Desmond simulations[7], (NPT ensemble (300 K, 1bar), SPC water, Langevin barostat), in each of the complexes under analysis (PDB IDs: 5G2B; 5G5V; 5L8Y; 5LAQ; 5LBO; 6HWO; 6GXQ; 6RFN; 6RFW).

Ligand docking

The ligands reported in SI Tables 1, 2, and 3 were docked into the TbrPDEB1 and hPDE4 structures using Glide[8 9] core-constrained docking with default parameters, using the X-ray ligand as reference (with the standard precision scoring function).

We then refined the alignment into the binding site of both PDE models for the subsequent FEP+ calculation using the Flexible Ligand Alignment tool[10] with the maximum common substructure algorithm.

FEP+ calculation

Free energy calculations were performed using the FEP+ method[11] with Schrödinger suite in the versions 2019-4 to 2020-1. Maps were generated with default settings (optimal topology).

The OPLS3e[12, 13] force field with custom parameters was used to predict the relative energy of binding of the ligands. A detailed description of the implementation of the methodology can be found elsewhere[14-16]. The missing torsions parameters of the ligands were computed using the Force Field Builder[17] tool.

Maps were generated with default settings (optimal topology). FEP+ jobs were run for 10 ns sampling time per λ-window for a total of 12 λ-windows. The output was analyzed with the FEP+ panel in Maestro**.** Replica exchange with solute Tempering (REST) was used as sampling method as reported in prior publications[16,18] (with the highest effective temperature of 1000 K for a typical perturbation with about 20 heavy atoms in the hot region)[16] The Bennett acceptance ratio method was used to calculate the free energy difference between neighboring λ windows. The simulations were performed at NPT ensemble (300 K, 1bar), using SPC water model.

Calculations were run on the GPU compute nodes with Dual Intel Xeon CPU E5-2620 v4 @2.10GHz (16 core total), and NVIDIA GeForce GTX1080 GPUs. For this study, we specifically ran the FEP+ simulations across 4 GPUs. The FEP+ calculation with the highest number of ligands in the map from Table 3 took almost 27 h to complete. It must be noted that the duration of FEP+ calculations is also dependent by the hardware used, the size of the system, the number of ligands, the number of lambda windows (default for the FEP+ implementation are as follows: 12 for neutral perturbations, 16 for core-hopping perturbations, and 24 for charge-hopping perturbations), and the length of the simulation.

Figure S1: Sequence alignment of human PDE4D, PDE4B, and parasite TbrPDEB1, colored by identity.

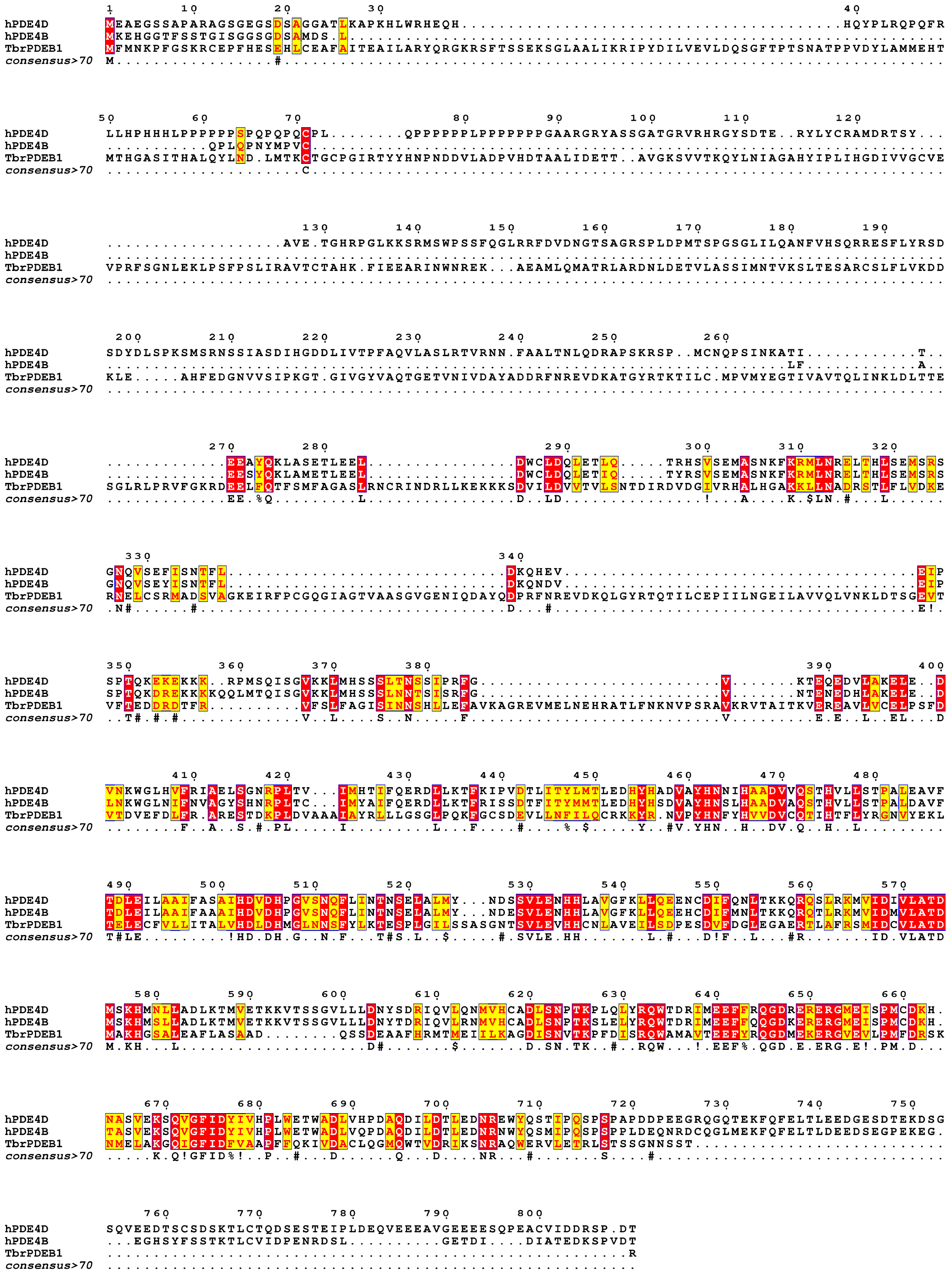

Figure S2. Interaction fingerprints and 2D ligand graphs

Interaction graphs and fingerprints of NPD-1335 co-crystallized in PDB IDs: 6GXQ (TbrPDEB1) and 6HWO (hPDE4D); NPD-038 co-crystallized in PDB ID: 5G5V (TbrPDEB1); NPD-008 co-crystallized in PDB ID: 5G2B (TbrPDEB1) and NPD-001 co-crystallized in PDB ID: 5LAQ (hPDE4B).

6GXQ (TbrPDEB1)

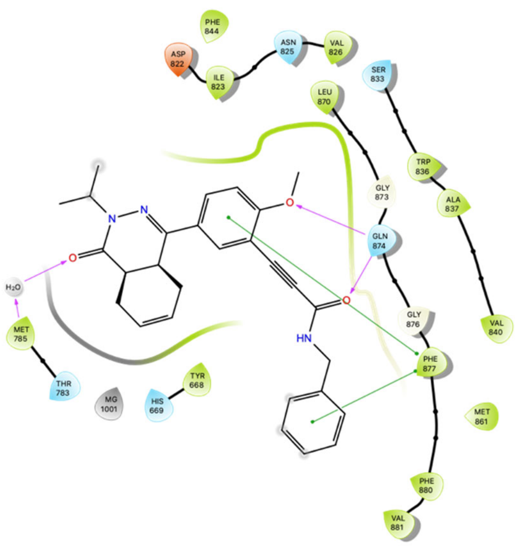

6HWO (hPDE4D)

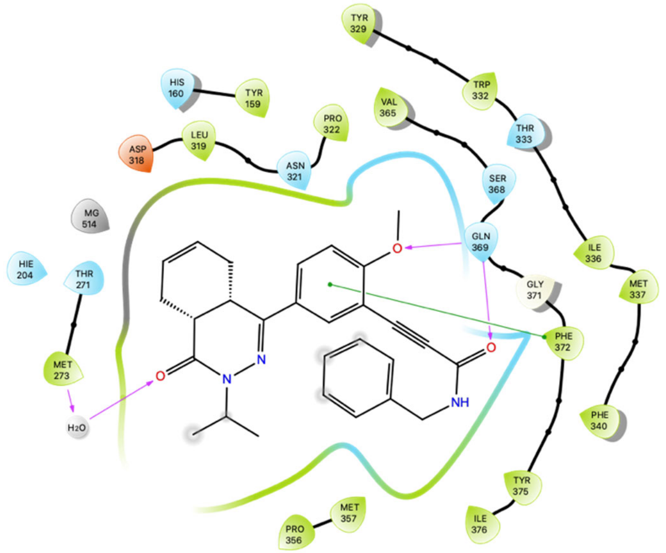

● Hydrophobic ● Aromatic face-to-face ● Aromatic face-to-edge ● H-bond donor ● H-bond receptor

	MB1	MB	HC1			Q1					Q2	HC	Q2	S		Q2	S			
hPDE4B1	21H 315	22D 318 (Hydrophobic)	23L 319 (Hydrophobic)	24S 320	25N 321 (Hydrophobic)	26P 322	27Y 329 (Hydrophobic)	28R 330	29W 332	30T 333 (Hydrophobic)	31D 334	32I 336 (Hydrophobic)	33M 337 (Hydrophobic)	34E 339	35F 340 (Hydrophobic)	36F 341	37Q 343	38S 355	39P 356	40M 357 (Hydrophobic)
TbrPDEB1	21K 819	22D 822 (Hydrophobic)	23I 823 (Hydrophobic)	24S 824	25N 825 (Hydrophobic)	26V 826	27S 833	28R 834	29W 836 (Hydrophobic)	30A 837 (Hydrophobic)	31M 838	32V 840 (Hydrophobic)	33T 841	34E 843	35F 844 (Hydrophobic)	36Y 845	37Q 847	38L 859	39P 860	40M 861 (Hydrophobic)

	S	Q2								Q	HC2	HC	Q1	HC2			Q1	Met1	Met2
hPDE4B1	41C 358	42N 362	43A 363	44- -	45S 364	46V 365	47E 366	48K 367	49S 368 (Hydrophobic, H-bond receptor)	50Q 369 (Hydrophobic, H-bond donor)	51G 371 (Hydrophobic)	52F 372 (Hydrophobic, Aromatic face-to-face, Aromatic face-to-edge)	53I 373	54Y 375	55I 376 (Hydrophobic)	56V 377	57Y 406	58X	59X
TbrPDEB1	41F 862	42K 866	43N 867	44M 868	45E 869	46L 870	47A 871	48K 872	49G 873 (Hydrophobic)	50Q 874 (Hydrophobic, H-bond donor)	51G 876 (Hydrophobic)	52F 877 (Hydrophobic, Aromatic face-to-face, Aromatic face-to-edge)	53I 878	54F 880	55V 881 (Hydrophobic)	56A 882	57W 911	58X	59X

5G5V (TbrPDEB1)

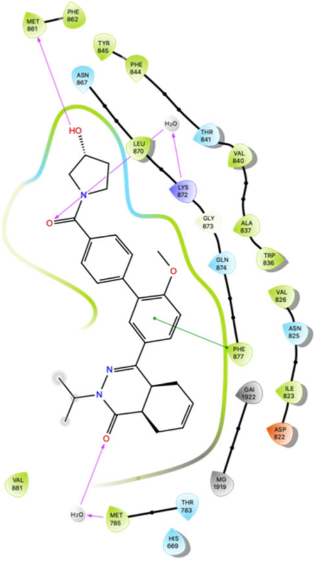

● Hydrophobic ● Aromatic face-to-face ● Aromatic face-to-edge ● H-bond donor ● H-bond receptor

MB1	MB	HC1			Q1					Q2	HC	Q2	S		Q2	S			
21K 819	22D 822	23I 823	24S 824	25N 825	26V 826	27S 833	28R 834	29W 836	30A 837	31M 838	32V 840	33T 841	34E 843	35F 844	36Y 845	37Q 847	38L 859	39P 860	40M 861
	Hydrophobic	Hydrophobic		Hydrophobic					Hydrophobic		Hydrophobic	Hydrophobic		Hydrophobic, Aromatic face-to-face	Hydrophobic				Hydrophobic

S	Q2								Q	HC2	HC	Q1	HC2			Q1	Met1	Met2
41F 862	42K 866	43N 867	44M 868	45E 869	46L 870	47A 871	48K 872	49G 873	50Q 874	51G 876	52F 877	53I 878	54F 880	55V 881	56A 882	57W 911	58X	59X
		Hydrophobic						Hydrophobic	Hydrophobic, H-bond donor		Hydrophobic, Aromatic face-to-face, Aromatic face-to-edge							

5G2B (TbrPDEB1)

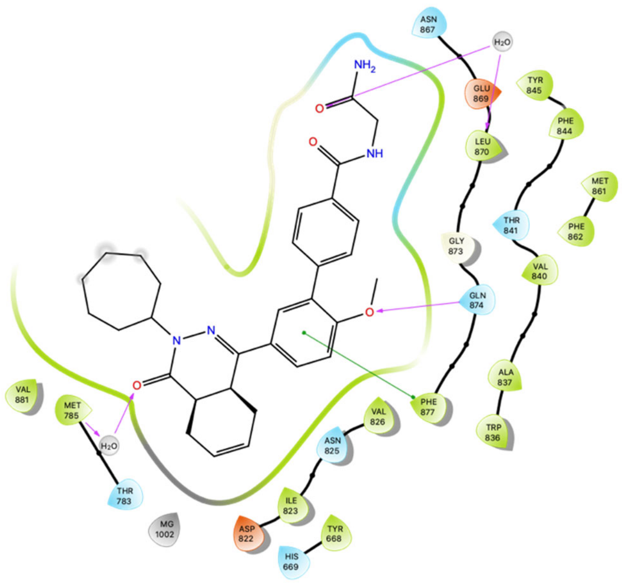

● Hydrophobic ● Aromatic face-to-face ● Aromatic face-to-edge ● H-bond donor ● H-bond receptor

MB1	MB	HC1			Q1					Q2	HC	Q2	S		Q2	S			
21K 819	22D 822	23I 823	24S 824	25N 825	26V 826	27S 833	28R 834	29W 836	30A 837	31M 838	32V 840	33T 841	34E 843	35F 844	36Y 845	37Q 847	38L 859	39P 860	40M 861
	● Hydrophobic	● Hydrophobic		● Hydrophobic					● Hydrophobic		● Hydrophobic	● Hydrophobic		● Hydrophobic<br>● Aromatic face-to-face	● Hydrophobic<br>● H-bond receptor				● Hydrophobic

S	Q2								Q	HC2	HC	Q1	HC2			Q1	Met1	Met2
41F 862	42K 866	43N 867	44M 868	45E 869	46L 870	47A 871	48K 872	49G 873	50Q 874	51G 876	52F 877	53I 878	54F 880	55V 881	56A 882	57W 911	58X	59X
		● Hydrophobic						● Hydrophobic	● Hydrophobic<br>● H-bond donor		● Hydrophobic<br>● Aromatic face-to-face<br>● Aromatic face-to-edge			● Hydrophobic				

5LAQ (hPDE4B)

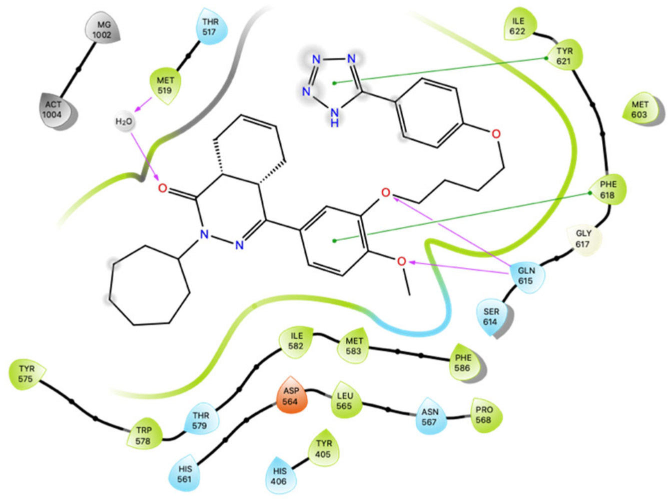

Hydrophobic, Aromatic face-to-face, Aromatic face-to-edge, H-bond donor, H-bond receptor

MB1	MB	HC1			Q1					Q2	HC	Q2	S		Q2	S			
21H 561	22D 564	23L 565	24S 566	25N 576	26P 568	27Y 575	28R 576	29W 578	30T 579	31D 580	32I 582	33M 583	34E 585	35F 586	36F 587	37Q 589	38S 601	39P 602	40M 603
	Hydrophobic	Hydrophobic		Hydrophobic		Hydrophobic		Hydrophobic	Hydrophobic		Hydrophobic	Hydrophobic		Hydrophobic					Hydrophobic

S	Q2								Q	HC2	HC	Q1	HC2			Q1	Met1	Met2
41C 604	42N 608	43A 609	44- -	45S 610	46V 611	47E 612	48K 613	49S 614	50Q 615	51G 617	52F 618	53I 619	54Y 621	55I 622	56V 623	57Y 652	58X	59X
								Hydrophobic	Hydrophobic, H-bond donor	Hydrophobic	Hydrophobic, Aromatic face-to-face		Hydrophobic, Aromatic face-to-face	Hydrophobic				

Table S1. Chemical structures of tetrahydrophthalazinones ligands of validation set 1, Glide SP score, ΔG values experimentally determined and respective ΔG computed by FEP+.

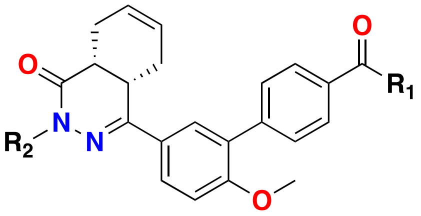

			TbrPDEB1			hPDE4		
Ligand ID	R_1	R_2	Glide SP score	Pred. ΔG	Exp. ΔG	Glide SP score	Pred. ΔG	Exp. ΔG
NPD-008	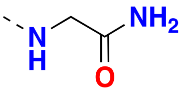	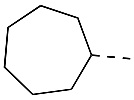	-11.75	-10.60	-10.23	-8.31	-7.67	-8.46
NPD-0734	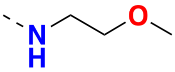	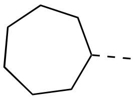	-7.13	-8.01	-9.41	-8.09	-7.93	-8.32
NPD-0800	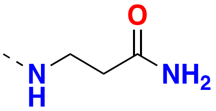	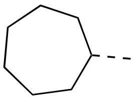	-11.09	-9.13	-9.00	-8.43	-8.51	-8.59
NPD-0936	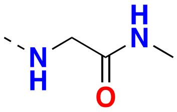	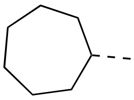	-11.41	-8.97	-8.73	-8.04	-8.24	-7.64
NPD-0935	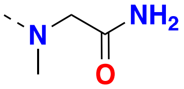	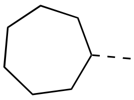	-10.45	-9.96	-8.73	-6.28	-7.83	-7.64
NPD-0937	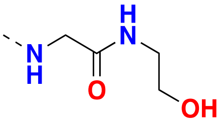	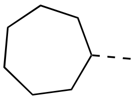	-11.94	-8.71	-8.73	-7.96	-7.61	-7.50
NPD-0878	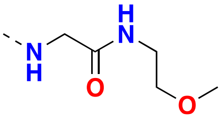	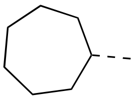	-11.98	-9.27	-8.59	-8.43	-7.71	-7.37
NPD-0801	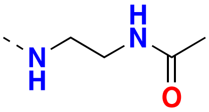	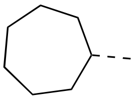	-11.00	-8.29	-8.59	-8.53	-7.85	-7.64
NPD-0942	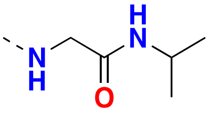	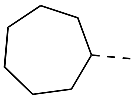	-10.87	-7.94	-7.78	-8.34	-8.07	-7.09

NPD-039	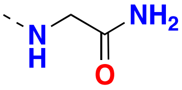	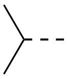	-10.90	-8.48	-9.55	-5.09	6.46	-7.78

Table S2. Chemical structures of tetrahydrophthalazinones ligands of validation set 2, Glide SP score, ΔG values experimentally determined and respective ΔG computed by FEP+.

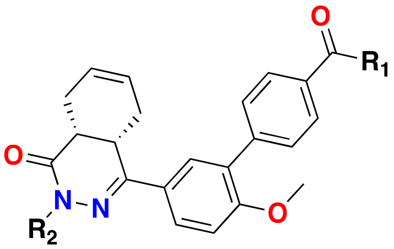

			TbrPDEB1			hPDE4		
Ligand ID	R_1	R_2	Glide SP score	Pred.ΔG	Exp.ΔG	Glide SP score	Pred.ΔG	Exp.ΔG
NPD-0746	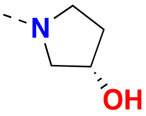	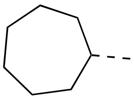	-13.17	-9.52	-9.55	-8.42	-7.82	-8.46
NPD-038	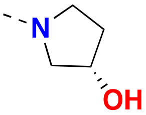	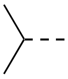	-12.61	-7.32	-9.14	-7.91	-6.92	-7.50
NPD-060	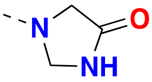	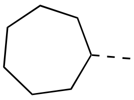	-11.76	-8.89	-8.46	-5.91	-7.90	-8.46
NPD-0887	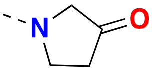	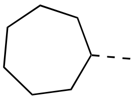	-10.78	-8.16	-8.19	-7.90	-7.37	-7.92
NPD-062	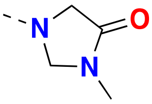	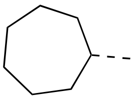	-10.29	-8.12	-7.50	-8.07	-7.99	-7.50
NPD-0802	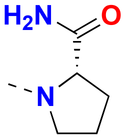	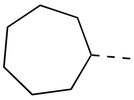	-10.06	-10.19	-9.00	-6.58	-7.77	-7.50
NPD-0885	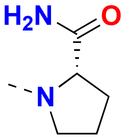	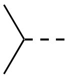	-9.68	-8.09	-8.46	-6.19	-7.29	-6.82

Table S3. Chemical structures of alkynamide phthalazinones ligands of validation set 3, Glide SP score, ΔG values experimentally determined and respective ΔG computed by FEP+.

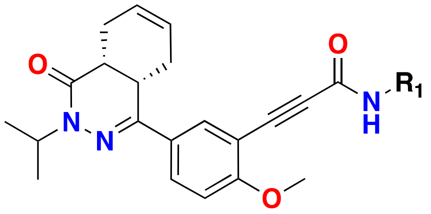

		TbrPDEB1			hPDE4D		
Ligand ID	R_1	Glide SP score	Pred.ΔG	Exp.ΔG	Glide SP score	Pred.ΔG	Exp.ΔG
NPD-1335	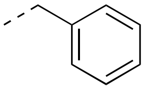	-9.65	-8.99	-9.30	-9.49	-8.95	-8.32
NPD-0361	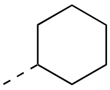	-8.15	-8.85	-8.50	-8.91	-9.28	-8.46
NPD-1016	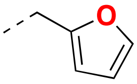	-8.48	-8.57	-9.40	-8.10	-8.18	-8.73
NPD-1018	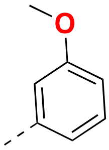	-8.30	-10.21	-9.15	-8.91	-10.66	-8.19
NPD-1024	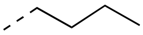	-7.84	-7.95	-8.92	-8.76	-8.24	-8.59
NPD-1038	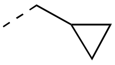	-7.86	-7.89	-8.68	-8.59	-7.97	-8.87
NPD-1039	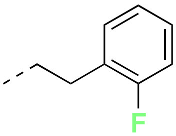	-8.16	-9.59	-8.87	-9.43	-9.01	-8.32
NPD-1041	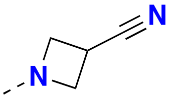	-7.28	-7.42	-8.40	-5.19	-8.16	-8.73
NPD-1042	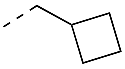	-8.09	-8.42	-8.91	-8.80	-8.54	-8.32
NPD-1168	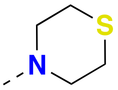	-8.25	-7.90	-8.25	-3.69	-8.28	-8.46

NPD-1169	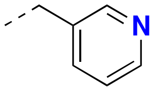	-8.60	-8.75	-9.52	-9.33	-8.44	-8.59
NPD-1171	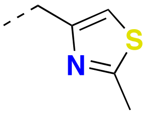	-8.58	-9.38	-8.92	-9-39	-8.90	-8.19
NPD-1174	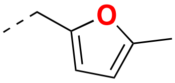	-8.81	-9.38	-9.15	-8.84	-8.49	-9.14
NPD-1319	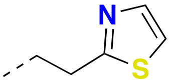	-8.85	-9.80	-8.96	-8.85	-8.02	-8.73
NPD-1320	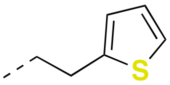	-9.01	-10.50	-8.76	-9.04	-8.84	-8.32
NPD-1321	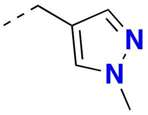	-8.29	-8.96	-9.39	-9.21	-8.48	-8.46
NPD-1322	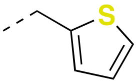	-8.66	-9.31	-9.35	-9.36	-9.17	-8.59
NPD-1323	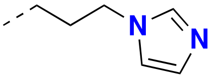	-7.94	-8.66	-8.72	-9.20	-7.92	-8.46
NPD-1334	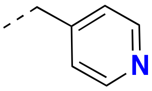	-8.47	-8.88	-9.56	-8.80	-8.59	-8.46
NPD-3153	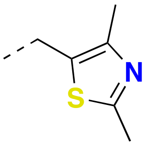	-8.66	-9.83	-9.13	-8.22	-9.31	-9.00
NPD-3155	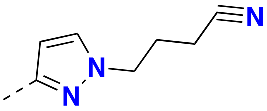	-8.45	-9.70	-8.53	-8.86	-8.95	-8.32
NPD-1043a	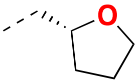	-7.94	-8.21	-8.69	-8.77	-7.07	-8.73
NPD-1043b	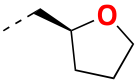	-8.20	-8.41	-8.69	-8.66	-7.54	-8.73

Table S4. Statistics on Data Sets used for FEP+ calculations

Protein system	PDB	Ligand set	No. ligands	Expected Exp. R^2	Expected FEP+ R^2	Predicted R^2	MUE (kcal/mol)	RMSE (kcal/mol)
**TbrPDEB1**	5G2B	1	13	0.74± 0.1	0.48±0.2	0.63	1.47± 0.3	2.05± 0.4
	5G2B[a]	1	10	0.45± 0.2	0.24±0.2	0.25	0.86± 0.1	1.03± 0.1
	5G2B	2	7	0.49± 0.2	0.28±0.2	0.37	1.70± 0.2	1.86± 0.3
	5G5V	2	9	0.43± 0.2	0.24±0.2	0.37	0.88± 0.1	1.03± 0.2
	6GXQ	3	23	0.17± 0.1	0.08±0.1	0.08	0.89± 0.1	1.08± 0.1
**hPDE4**	5LAQ	1	12	0.49± 0.2	0.26±0.2	0.45	0.69± 0.1	0.90± 0.2
	5LAQ	2	7	0.49± 0.2	0.28±0.2	0.15	0.76± 0.2	0.98± 0.3
	6HWO	3	23	0.10± 0.1	0.06±0.1	0.12	0.82± 0.1	1.00± 0.1

[a]Thr841 included in the REST region

Table S5. Flexibility and stability analysis of the X-ray structures in complex with PDEs inhibitors

Ligand ID	Species	PDB ID	RMSD Ligand[a] Å	RMSD FP[b] Å	RMSD PP[c] Å
**NPD-008**	TbrPDEB1	5G2B	1.8	0.34	3.53
**NPD-937**	TbrPDEB1	5L8Y	3.5	0.50	1.49
**NPD-038**	TbrPDEB1	5G5V	3.5	0.70	3.48
**NPD-001**	hPDE4B	5LAQ	1.8	0.62	1.08
	hPDE4D	5LBO	2.4	0.32	0.67
**NPD-1335**	hPDE4D	6HWO	/	0.27	0.78
	TbrPDEB1	6GXQ	/	0.26	0.89

[a] RMSD of the ligands measured during the MD simulation.[b] RMSD of on the protein backbone between various apo-forms (PDB IDs: 4I15, 4WZI and 3SL3 for TbrPDEB1, hPDE4B and hPDE4D, respectively) and holo-forms (PDB IDs: 5G2B; 5G57; 6GXQ for TbrPDEB1; PDB IDs: 5LAQ for hPDE4B; PDB IDs: 5LBO; 6HWO for hPDE4D) measured on the full protein (FP) and [c] on the P-pocket region (PP).

Table S6. Convergence data from FEP+ calculation

Convergence criteria fully integrated in the graphical interface of the FEP+ implementation are programmatically assessed and sorted in 3 categories: Good convergence rate: < 0.3 kcal mol^{-1} ns^{-1}; Fair convergence rate: 0.3 kcal mol^{-1} ns^{-1}; Bad convergence rate: > 0.3 kcal mol^{-1} ns^{-1}. Below are reported the number of edges and the associated convergence categories of FEP+ perturbations calculated for set 1, 2 and 3.

Species	Lig. Set	PDB	N. Total Edges	N. Edges with Good Convergence rate	N. Edges with Fair Convergence rate	N. Edges with Bad Convergence rate
**TbrPDEB1**	Set1	5G2B	16	0	14	2
	Set1	5G2B (REST)[a]	17	17	0	0
	Set2	5G2B	10	10	0	0
	Set2	5G5V	9	8	1	0
	Set3	6GXQ	34	30	4	0
**hPDE4**	Set1	5LAQ	18	15	3	0
	Set2	5LAQ	12	12	0	0
	Set3	6HWO	35	33	2	0

[a] Thr841 included in the REST region.

Figure S3: Examples of Good, Fair, and Bad convergence plots and the associated maps.

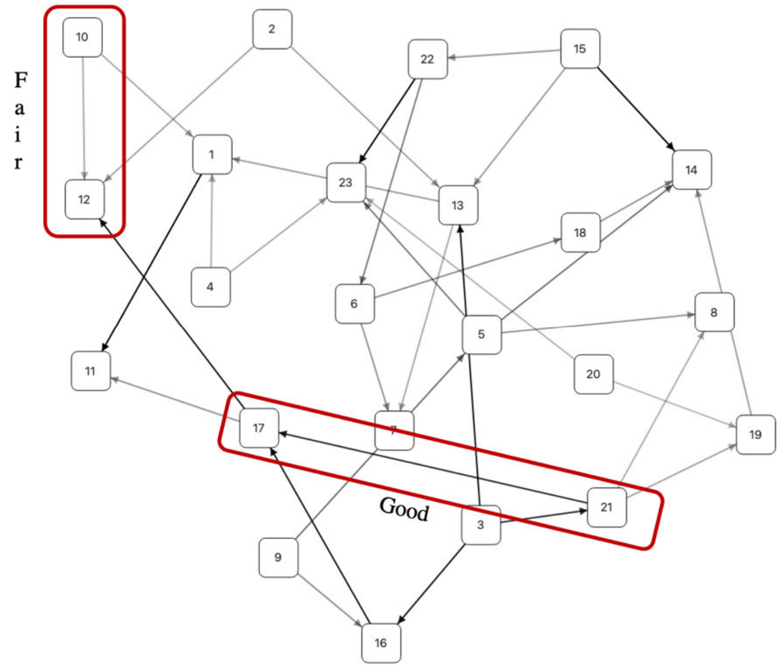

Complex Leg – Good Convergence 21 ->17

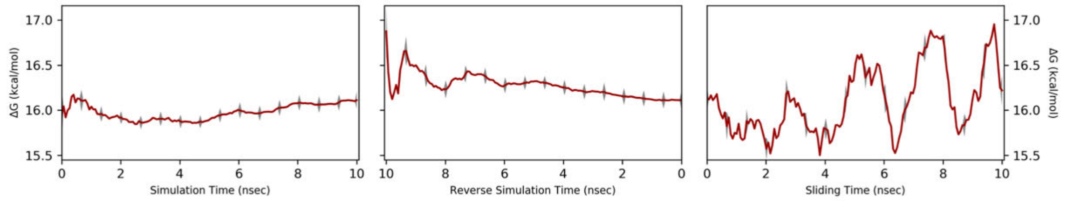

Solvent Leg – Good Convergence 21 ->17

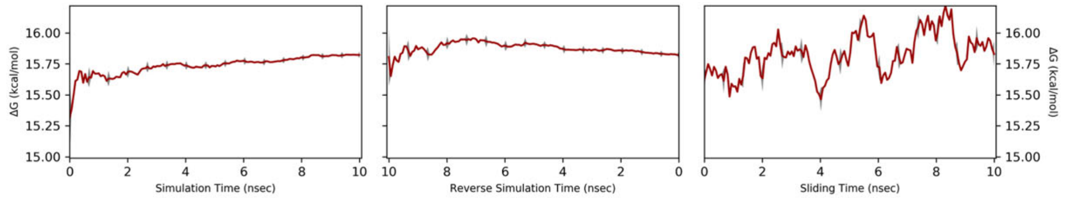

Complex Leg – Fair Convergence 10 ->12

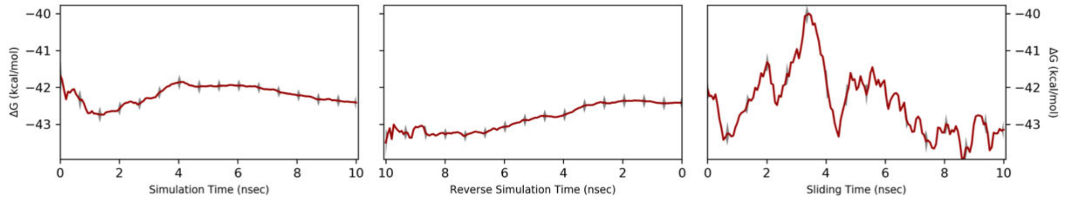

Solvent Leg – Fair Convergence 10 ->12

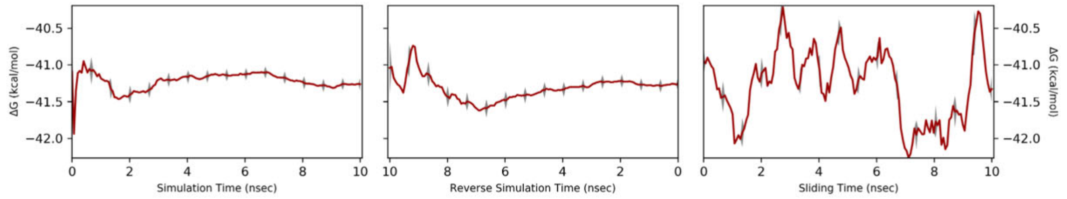

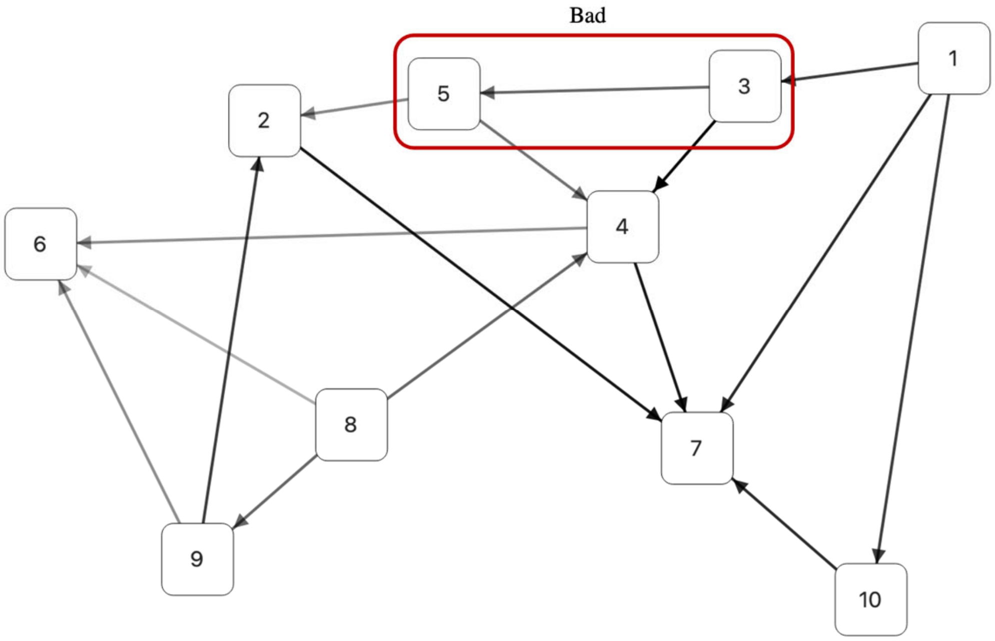

Complex Leg – Bad Convergence 3 ->5

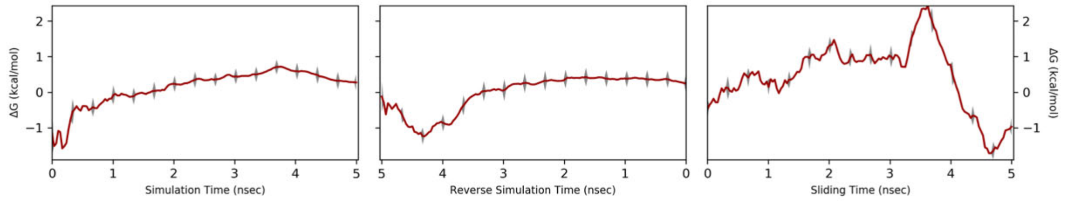

Solvent Leg – Bad Convergence 3 ->5

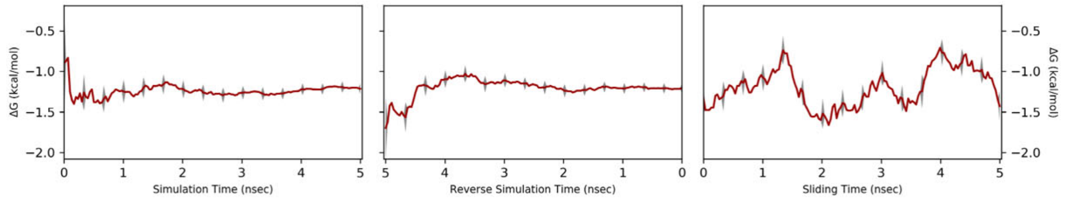

References

1. Schrödinger Release 2021-1: Maestro, Schrödinger, LLC, New York, NY, 2021.
2. *Schrödinger Release 2019- 4: LigPrep.*
3. Blaazer, A. R.; Singh, A. K.; de Heuvel, E.; Edink, E.; Orrling, K. M.; Veerman, J. J. N.; van den Bergh, T.; Jansen, C.; Balasubramaniam, E.; Mooij, W. J.; Custers, H.; Sijm, M.; Tagoe, D. N. A.; Kalejaiye, T. D.; Munday, J. C.; Tenor, H.; Matheeussen, A.; Wijtmans, M.; Siderius, M.; de Graaf, C.; Maes, L.; de Koning, H. P.; Bailey, D. S.; Sterk, G. J.; de Esch, I. J. P.; Brown, D. G.; Leurs, R., Targeting a Subpocket in Trypanosoma brucei Phosphodiesterase B1 (TbrPDEB1) Enables the Structure-Based Discovery of Selective Inhibitors with Trypanocidal Activity. *J Med Chem* **2018,** *61* (9), 3870-3888.
4. de Heuvel, E.; Singh, A. K.; Boronat, P.; Kooistra, A. J.; van der Meer, T.; Sadek, P.; Blaazer, A. R.; Shaner, N. C.; Bindels, D. S.; Caljon, G.; Maes, L.; Sterk, G. J.; Siderius, M.; Oberholzer, M.; de Esch, I. J. P.; Brown, D. G.; Leurs, R., Alkynamide phthalazinones as a new class of TbrPDEB1 inhibitors (Part 2). *Bioorg Med Chem* **2019,** *27* (18), 4013-4029.
5. Sastry, G. M.; Adzhigirey, M.; Day, T.; Annabhimoju, R.; Sherman, W., Protein and ligand preparation: parameters, protocols, and influence on virtual screening enrichments. *J Comput Aided Mol Des* **2013,** *27* (3), 221-34.
6. Schrödinger Release 2021-1: Protein Preparation Wizard; Epik, Schrödinger, LLC, New York, NY, 2021; Impact, Schrödinger, LLC, New York, NY; Prime, Schrödinger, LLC, New York, NY, 2021.
7. Schrödinger Release 2021-1: Desmond Molecular Dynamics System, D. E. Shaw Research, New York, NY, 2021. Maestro-Desmond Interoperability Tools, Schrödinger, New York, NY, 2021.
8. Schrödinger Release 2021-1: Glide, Schrödinger, LLC, New York, NY, 2021.
9. Friesner, R. A.; Murphy, R. B.; Repasky, M. P.; Frye, L. L.; Greenwood, J. R.; Halgren, T. A.; Sanschagrin, P. C.; Mainz, D. T., Extra precision glide: docking and scoring incorporating a model of hydrophobic enclosure for protein-ligand complexes. *J Med Chem* **2006,** *49* (21), 6177-96.
10. Friesner, R. A.; Banks, J. L.; Murphy, R. B.; Halgren, T. A.; Klicic, J. J.; Mainz, D. T.; Repasky, M. P.; Knoll, E. H.; Shelley, M.; Perry, J. K.; Shaw, D. E.; Francis, P.; Shenkin, P. S., Glide: a new approach for rapid, accurate docking and scoring. 1. Method and assessment of docking accuracy. *J Med Chem* **2004,** *47* (7), 1739-49.
11. Schrödinger Release 2021-1: FEP+, Schrödinger, LLC, New York, NY, 2021.
12. Harder, E.; Damm, W.; Maple, J.; Wu, C.; Reboul, M.; Xiang, J. Y.; Wang, L.; Lupyan, D.; Dahlgren, M. K.; Knight, J. L.; Kaus, J. W.; Cerutti, D. S.; Krilov, G.; Jorgensen, W. L.; Abel, R.; Friesner, R. A., OPLS3: A Force Field Providing Broad Coverage of Drug-like Small Molecules and Proteins. *J Chem Theory Comput* **2016,** *12* (1), 281-96.
13. Shivakumar, D.; Harder, E.; Damm, W.; Friesner, R. A.; Sherman, W., Improving the Prediction of Absolute Solvation Free Energies Using the Next Generation OPLS Force Field. *J Chem Theory Comput* **2012,** *8* (8), 2553-8.
14. Yu, H. S.; Deng, Y.; Wu, Y.; Sindhikara, D.; Rask, A. R.; Kimura, T.; Abel, R.; Wang, L., Accurate and Reliable Prediction of the Binding Affinities of Macrocycles to Their Protein Targets. *J Chem Theory Comput* **2017,** *13* (12), 6290-6300.
15. Kuhn, B.; Tichy, M.; Wang, L.; Robinson, S.; Martin, R. E.; Kuglstatter, A.; Benz, J.; Giroud, M.; Schirmeister, T.; Abel, R.; Diederich, F.; Hert, J., Prospective Evaluation of Free Energy Calculations for the Prioritization of Cathepsin L Inhibitors. *J Med Chem* **2017,** *60* (6), 2485-2497.
16. Abel, R.; Wang, L.; Harder, E. D.; Berne, B. J.; Friesner, R. A., Advancing Drug Discovery through Enhanced Free Energy Calculations. *Acc Chem Res* **2017,** *50* (7), 1625-1632.

17. Shivakumar, D.; Williams, J.; Wu, Y.; Damm, W.; Shelley, J.; Sherman, W., Prediction of Absolute Solvation Free Energies using Molecular Dynamics Free Energy Perturbation and the OPLS Force Field. *J Chem Theory Comput* **2010,** *6* (5), 1509-19.

18. Wang, L.; Deng, Y.; Knight, J. L.; Wu, Y.; Kim, B.; Sherman, W.; Shelley, J. C.; Lin, T.; Abel, R., Modeling Local Structural Rearrangements Using FEP/REST: Application to Relative Binding Affinity Predictions of CDK2 Inhibitors. *J Chem Theory Comput* **2013,** *9* (2), 1282-93.
